# Severe Darier disease complicated by recurrent multidrug-resistant cutaneous infection

**DOI:** 10.1016/j.jdcr.2026.06.010

**Published:** 2026-06-13

**Authors:** Ivana Ho, Elizabeth W. Beiermann, Alecia R. Anyim, Jack B. Newcomer, Marian T. McEvoy

**Affiliations:** a Division of Hospital Internal Medicine, Mayo Clinic, Rochester, Minnesota; b Division of Dermatology, Mayo Clinic, Rochester, Minnesota

**Keywords:** bacteriophage therapy, cutaneous dysbiosis, Darier disease, genodermatosis, keratosis follicularis, multidrug-resistant infection

## Case description

A 39-year-old woman with severe Darier disease (DD) and a history of multiple hospitalizations for recurrent multidrug resistant cutaneous infections presented with painful, worsening lesions involving the gluteal and perineal regions. Acitretin was discontinued 2 months prior when prescribers were unable to continue refills in absence of updated laboratory monitoring, resulting in progressive cutaneous disease. She had been treated intermittently with acitretin since adolescence and had previously achieved near-complete disease control while receiving therapy.

**Fig 1:**
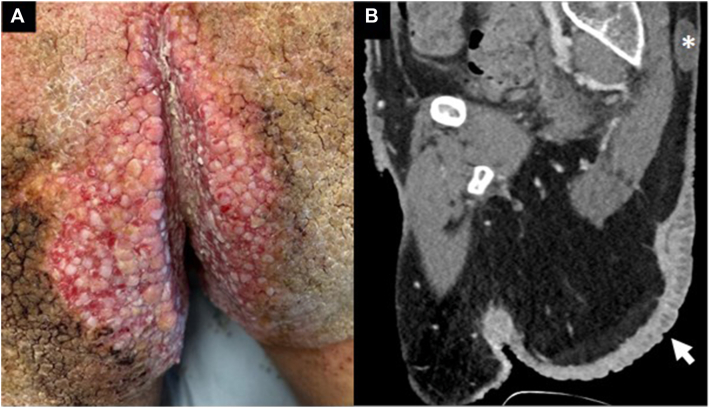
**A,** Erythematous, hyperkerotic plaques with erosion and purulent drainage involving the gluteal cleft consistent with secondary infection. **B,** Computed tomography of the pelvis demonstrating a gluteal abscess (∗) and thickened hyperkeratotic skin consistent with Darier disease (*arrow*).

**Fig 2:**
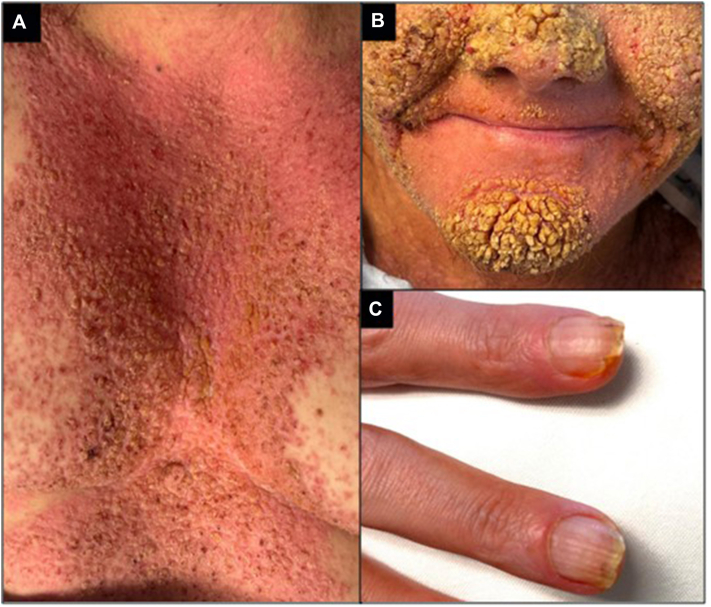
**A,** Numerous hyperkeratotic popules and plaques on the chest. **B,** Hyperkeratotic popules and plaques involving the face, demonstrating classic seborrheic distribution. **C,** Nail abnormalities demonstrating longitudinal ridging and characteristic V-shaped distal notching.

Examination revealed extensive erythematous, hyperkeratotic plaques with erosions and purulent drainage in intertriginous areas (Fig 1, *A*), along with widespread seborrheic distribution of keratotic papules on the face, chest, and back (Fig 2, *A* and *B*). Nail findings showed longitudinal ridging with V-shaped distal notching (Fig 2, *C*). Imaging demonstrated a gluteal abscess (Fig 1, *B*). Wound cultures grew polymicrobial organisms, including carbapenem-resistant *Pseudomonas aeruginosa*, methicillin-resistant *Staphylococcus aureus*, *Enterococcus faecalis*, and *Proteus mirabilis*.

The abscess was drained and treated with intravenous antibiotics. Dermatologic therapy included reinitiation of acitretin, keratolytics, and antiseptic soaks. Due to persistent infection with progressively resistant organisms despite culture-directed antimicrobial therapy, adjunctive investigational bacteriophage therapy was initiated. The patient improved clinically with decreased drainage, pain, and leukocytosis following multidisciplinary management.

**Question: Which of the following interventions is most important in preventing recurrent severe infections in patients with Darier disease?**

**A.** Chronic suppressive antibiotics
**B.** Long-term systemic corticosteroids
**C.** Maintenance systemic retinoid therapy
**D.** Routine antiviral prophylaxis
**E.** Daily antiseptic washes alone

Answer: **C.** Maintenance systemic retinoid therapy.

## Discussion

DD is a chronic genodermatosis caused by mutations in ATP2A2, resulting in impaired keratinocyte adhesion and epidermal barrier dysfunction, predisposing patients to bacterial and viral infections.^1^

Systemic retinoids remain the cornerstone of therapy for moderate-to-severe DD because they improve keratinocyte differentiation and help restore epidermal barrier integrity.^1^^,^^2^ In this patient, cessation of acitretin preceded rapid clinical deterioration and recurrent polymicrobial multidrug resistant infection, highlighting the importance of uninterrupted maintenance retinoid therapy in preventing severe infectious complications. Clinical studies demonstrate substantial improvement in most patients with oral retinoids, and long-term observational data support their sustained efficacy with maintenance use.^2^^,^^3^ This aligns with the patient’s prior history of disease control while taking acitretin. Emerging evidence suggests that biologic therapies targeting the IL-17 and IL-23 pathway may benefit select patients with severe refractory disease and may offer an alternative for patients who are unable to tolerate or maintain long-term retinoid therapy. However, current evidence is limited to small case series and case reports, and systemic retinoids remain the standard fist-line treatment.^1^

Patients with DD also frequently exhibit cutaneous dysbiosis with increased colonization of organisms, such as *S. aureus*, which is also correlated with increased disease severity.^4^ Although antimicrobial therapies and topical antiseptics may temporarily reduce microbial burden, they do not correct the underlying epidermal barrier defect that drives recurrent infection and may contribute to antimicrobial resistance.^5^ Our patient ultimately required adjunctive investigational bacteriophage therapy after failure of conventional antimicrobial strategies. Uncontrolled epidermal barrier disease may promote persistent polymicrobial colonization, while repeated antimicrobial exposure may contribute to selection for resistant organisms, illustrating the complex interplay between barrier dysfunction and microbial overgrowth.^4^^,^^5^

Ultimately, sustained control of the underlying epidermal barrier defect with maintenance systemic retinoid therapy helps reduce the risk of recurrent severe infections in DD.

### Declaration of generative AI and AI-assisted technologies in the writing process

During the preparation of this work the authors used ChatGPT to edit the paper. After using this tool, the authors reviewed and edited the content and take full responsibility for the content of the published article.

## Conflicts of interest

The authors report no financial conflicts of interest. Investigational bacteriophages used during this patient’s care were provided by Qeen Biotechnologies Inc. as part of a separate clinical protocol. Drs Gina Suh and Nancy Tawil had no role in the preparation of this manuscript.
